# Diagnostic value of diffusion kurtosis imaging for brain injury in term neonates with hypoxic–ischemic encephalopathy

**DOI:** 10.3389/fnins.2026.1806571

**Published:** 2026-05-26

**Authors:** Xueyuan Wang, Xianglong Liu, Penghua Zhang, Wuzi Xu, Qingna Xing, Meiying Cheng, Xuefei Xu, Xueyao Yang, Xiaoan Zhang, Xin Zhao

**Affiliations:** 1 Department of Medical Imaging, Third Affiliated Hospital of Zhengzhou University, Zhengzhou, Henan, China; 2 Tianjian Laboratory of Advanced Biomedical Sciences, Institute of Advanced Biomedical Sciences, Zhengzhou University, Zhengzhou, Henan, China; 3 Clinical Medical College of Henan University of Science and Technology, Luoyang, Henan, China; 4 Department of Cardiology, Henan Provincial Chest Hospital, Zhengzhou, Henan, China; 5 Toxicology Department, Center for Drug Safety Evaluation and Research of Zhengzhou University, Zhengzhou, Henan, China

**Keywords:** diffusion kurtosis imaging, neonatal brain injury, hypoxic-ischemic encephalopathy, severity stratification, term neonates

## Abstract

**Background:**

Neonatal hypoxic–ischemic encephalopathy (HIE) stands as a primary cause of neonatal brain injury, mortality, and long-term neurodevelopmental impairment, imposing a considerable burden on public health systems worldwide. Despite advances in clinical practice, accurate early diagnosis and reliable severity stratification of HIE remain formidable challenges when relying solely on conventional neuroimaging modalities.

**Materials and methods:**

A cohort of 66 term neonates with clinically confirmed HIE and 25 age-matched healthy controls underwent cranial magnetic resonance imaging (MRI) combined with diffusion kurtosis imaging (DKI) within the first 28 days of life. A region-of-interest (ROI)-based quantitative analysis was conducted to quantify kurtosis and diffusion metrics across distinct white matter and deep gray matter regions. The diagnostic utility and severity-stratifying capacity of these DKI parameters were evaluated using receiver operating characteristic (ROC) curve analysis, while their potential correlations with 1-min and 5-min Apgar scores were further explored.

**Results:**

ROI-based analysis revealed significant differences in DKI parameters between neonates with HIE and healthy controls across multiple brain regions. Notably, DKI parameters derived from deep gray matter exhibited superior diagnostic performance and severity stratification capacity, with several kurtosis indices achieving an area under the ROC curve (AUC) greater than 0.90. Subgroup comparisons further demonstrated that specific DKI parameters differed significantly between mild and moderate, and between mild and moderate-to-severe HIE cases. Among all measured indices, the mean kurtosis of the corpus callosum yielded relatively high discriminative efficacy. Additionally, a subset of DKI parameters showed significant correlations with Apgar scores, with a greater number of parameters correlating strongly with the 5-min Apgar score.

**Conclusion:**

DKI yields robust quantitative biomarkers that facilitate the accurate diagnosis and reliable severity stratification of neonatal HIE, with deep gray matter emerging as a pivotal indicator of brain injury severity. These findings underscore the potential value of advanced diffusion-weighted imaging techniques in enabling early risk stratification and standardized assessment of neonatal brain injury, which carries profound implications for the optimization of public health-oriented neonatal care strategies.

## 1 Introduction

Neonatal hypoxic–ischemic encephalopathy (HIE) is a leading cause of perinatal brain injury and a major contributor to cerebral palsy, childhood mortality, and long-term neurodevelopmental disability worldwide. With an estimated incidence of approximately 1.5 per 1,000 live births (Greco et al., 2020; Kurinczuk et al., 2010; Smith et al., 2000), HIE imposes a substantial and enduring burden on affected children, their families, and public health systems.

HIE results from insufficient oxygen and blood supply to the developing brain during the perinatal period, most commonly due to perinatal asphyxia occurring before, during, or shortly after delivery. Brain injury may be focal or diffuse and tends to involve regions with high metabolic demand or limited perfusion reserve, including watershed areas, the thalamus, and the hippocampus. In cases of severe global hypoxia, diffuse cerebral edema may occur (Chao et al., 2006; Bobba et al., 2023). Injury patterns are closely associated with the severity and duration of hypoxic–ischemic insult as well as the maturational stage of the neonatal brain. Compared with preterm infants, term and near-term neonates are particularly vulnerable owing to higher metabolic activity in myelinated and pre-myelinated fibers (Bano et al., 2017).

In clinical practice, the Apgar score is widely used for the initial assessment of newborns immediately after birth and helps identify infants at increased risk of hypoxic–ischemic injury. Lower Apgar scores, particularly at 5 min, are consistently associated with a higher risk of HIE and adverse neurological outcomes (Debillon et al., 2023; Chen et al., 2023). However, Apgar scoring provides limited information on the extent and spatial distribution of brain injury and does not reliably reflect underlying microstructural damage.

Magnetic resonance imaging (MRI) is regarded as the most sensitive imaging modality for evaluating suspected neonatal HIE and is routinely used to assess injury presence and patterns. Conventional MRI sequences, however, rely largely on qualitative visual assessment and remain limited in their ability to provide objective and reproducible quantitative biomarkers for early injury characterization and severity stratification, both of which are essential for informed clinical decision-making. Advanced diffusion-based MRI techniques have been explored to address the limitations of conventional imaging in neonatal HIE. Diffusion tensor imaging (DTI) enables quantitative assessment of white matter integrity and has demonstrated value in early diagnosis and outcome prediction (Seo et al., 2017; Tusor et al., 2012). However, DTI assumes that water diffusion follows a Gaussian distribution, an assumption that does not adequately capture the complex and heterogeneous microenvironment of biological tissues, particularly in the developing neonatal brain.

Diffusion kurtosis imaging (DKI) extends the DTI framework by characterizing non-Gaussian diffusion behavior and provides additional information on tissue microstructural complexity and diffusion heterogeneity. By capturing subtle alterations in diffusion restriction, DKI has the potential to detect microstructural injury that may not be apparent on conventional diffusion metrics (Jensen and Helpern, 2010). Despite these advantages, clinical application of DKI in neonatal HIE remains limited, partly due to technical challenges and the scarcity of well-characterized clinical cohorts (Gao et al., 2016; Xiao et al., 2020; He et al., 2020; Sun et al., 2021; Wu et al., 2018).

Previous experimental and preclinical studies have provided important insights into the potential utility of DKI in hypoxic–ischemic brain injury. For example, Gao et al. (2016) reported that DKI-derived parameters distinguished complex hypoxic–ischemic lesions from isolated T2 hyperintensities in neonatal white matter, whereas conventional DTI metrics did not. Similarly, studies using neonatal piglet models have shown that DKI can detect microstructural abnormalities at very early time points after injury and may better delineate lesion extent and evolution compared with diffusion-weighted imaging (Xiao et al., 2020; He et al., 2020; Han et al., 2023). Despite these encouraging findings, most existing evidence is derived from animal models or small-scale studies, and systematic clinical validation in human neonates remains limited. In particular, the diagnostic performance of DKI across different brain regions, its ability to stratify injury severity, and its associations with early clinical indicators such as Apgar scores have not been comprehensively evaluated in term neonates with HIE.

In this context, the present study evaluated the diagnostic and severity-stratification performance of DKI in term neonates with HIE. Using region-of-interest (ROI)-based quantitative analyses of white matter and deep gray matter structures, we compared DKI-derived parameters between neonates with HIE and healthy controls and examined their associations with 1-min and 5-min Apgar scores. By identifying sensitive, region-specific imaging markers of brain injury severity, this study aims to support earlier risk stratification and more objective, standardized assessment of neonatal HIE at the population level, with potential implications for timely clinical decision-making and long-term child neurological health.

## 2 Materials and methods

### 2.1 Participants

A total of 66 term and near-term neonates clinically diagnosed with HIE who underwent brain MRI at the Department of Medical Imaging of our hospital were retrospectively enrolled as the HIE group. This cohort comprised 35 males and 31 females, with a gestational age ranging from 36 to 40 weeks and a birth weight of 2,500–3,800 g. Infants with moderate and severe HIE in this cohort received standard therapeutic hypothermia per international neonatal HIE clinical guidelines, initiated within 6 h of birth (selective head cooling, core temperature maintained at 34.5 °C for 72 h followed by gradual rewarming), combined with conventional supportive care including hemodynamic stabilization, anticonvulsant therapy, glycemic control and respiratory support. Infants with mild HIE were not given hypothermia treatment, only receiving routine clinical monitoring and basic supportive care. The consistency of all therapeutic regimens was strictly standardized and controlled as a confounding factor in this study.

Inclusion criteria (Azzopardi et al., 2008) were based on previously established diagnostic standards: (1) gestational age ≥ 36 weeks; (2) documented evidence of fetal intrauterine distress or perinatal asphyxia; and (3) the presence of at least one of the following conditions: Apgar score ≤ 5 within 10 min after birth, requirement for resuscitation beyond 10 min, umbilical artery or capillary pH < 7.00 within 60 min after birth, or a base deficit ≥ 16 mmol/L within 60 min after birth. According to Sarnat and Sarnat (1976), classification system, neonates with HIE were further stratified into mild (*n* = 35), moderate (*n* = 27) and severe (*n* = 4) subgroups based on clinical neurological manifestations.

During the same study period, 25 neonates with normal neurological examinations and unremarkable brain MRI findings were recruited as healthy controls. The control group neonates underwent cranial MRI for non-neurological indications (e.g., ophthalmic disorders, facial subcutaneous hemangiomas, facial fractures). Their normal neurological status was jointly confirmed by two senior neuroradiologists (≥10 years of experience) and one attending neonatologist, based on unremarkable cranial MRI/DKI findings, normal neurological physical examinations (conducted within 28 days after birth), no perinatal distress or asphyxia, Apgar scores ≥8 at 1 and 5 min, umbilical artery blood pH > 7, and no history of central nervous system or severe somatic diseases.

No significant differences were observed between groups with respect to sex, gestational age, birth weight, or age at MRI examination (all *p* > 0.05) (Table 1).

**Table 1:** Demographic and clinical data of subjects.

Basic information	Groups				*x* ^2^	*p*
	Control group	Mild group	Moderate group	Severe group		
Gender (male/female)	19/8	19/16	14/13	2/2	–	0.496
Mode of delivery (cesarean section/vaginal delivery)	18/9	27/8	18/9	2/2	–	0.530
Gestational age at birth (weeks)	37.663 ± 1.483	37.371 ± 1.328	36.778 ± 1.027	36.643 ± 0.601	7.377	0.061
Birth weight (g)	2941.600 ± 433.154	2804.286 ± 300.022	2790.000 ± 341.693	2560.000 ± 94.163	7.196	0.066
Body weight at examination (g)	3734.000 ± 586.607	3528.57 ± 650.231	3456.296 ± 628.718	3025.000 ± 370.810	7.356	0.061
Postnatal age at examination (days)	17.600 ± 9.640	14.430 ± 8.631	10.740 ± 7.538	12.000 ± 10.100	5.768	0.123

Fisher’s exact test was adopted for statistical analysis of gender and mode of delivery. The Kruskal–Wallis test was employed for statistical analysis of gestational age at birth, birth weight, body weight at examination, and postnatal age at examination.

### 2.2 MRI acquisition

All neonates received intramuscular phenobarbital (5 mg/kg) approximately 30 min prior to MRI to minimize motion artifacts. MRI examinations were performed within the first 28 days of life. Throughout the scan, neonates were continuously monitored, and written informed consent was obtained from a legal guardian. The study protocol was approved by the hospital ethics committee (Approval No. 2024–369-01).

Imaging was conducted using a 3.0-T MRI scanner (SIGNA™ Pioneer, GE Healthcare, United States). The protocol included routine cranial MRI sequences and DKI. DKI acquisition parameters were as follows: 25 diffusion-encoding directions; TR = 8,200 ms; TE = minimum; slice thickness = 4 mm; interslice gap = 0 mm; FOV = 200 × 200 mm^2^; and b-values of 0, 1,000, and 2,000 s/mm^2^. The total DKI acquisition time was 7 min 23 s.

### 2.3 DKI processing and ROI analysis

All raw DKI data in DICOM format were converted to NIFTI format using the dcm2niix tool^1^ implemented in MRIcroGL. Head motion and eddy current correction were then performed using the FDT module in FSL Wiki,^2^ with the *b* = 0 image serving as the reference. Subsequently, parametric maps were calculated using DKE software (Diffusion Kurtosis Estimator).^3^ Diffusion and kurtosis metrics were generated, including fractional anisotropy (FA), mean diffusivity (MD), axial diffusivity (Da), and radial diffusivity (Dr), mean kurtosis (MK), axial kurtosis (Ka), and radial kurtosis (Kr).

**Figure 1:**
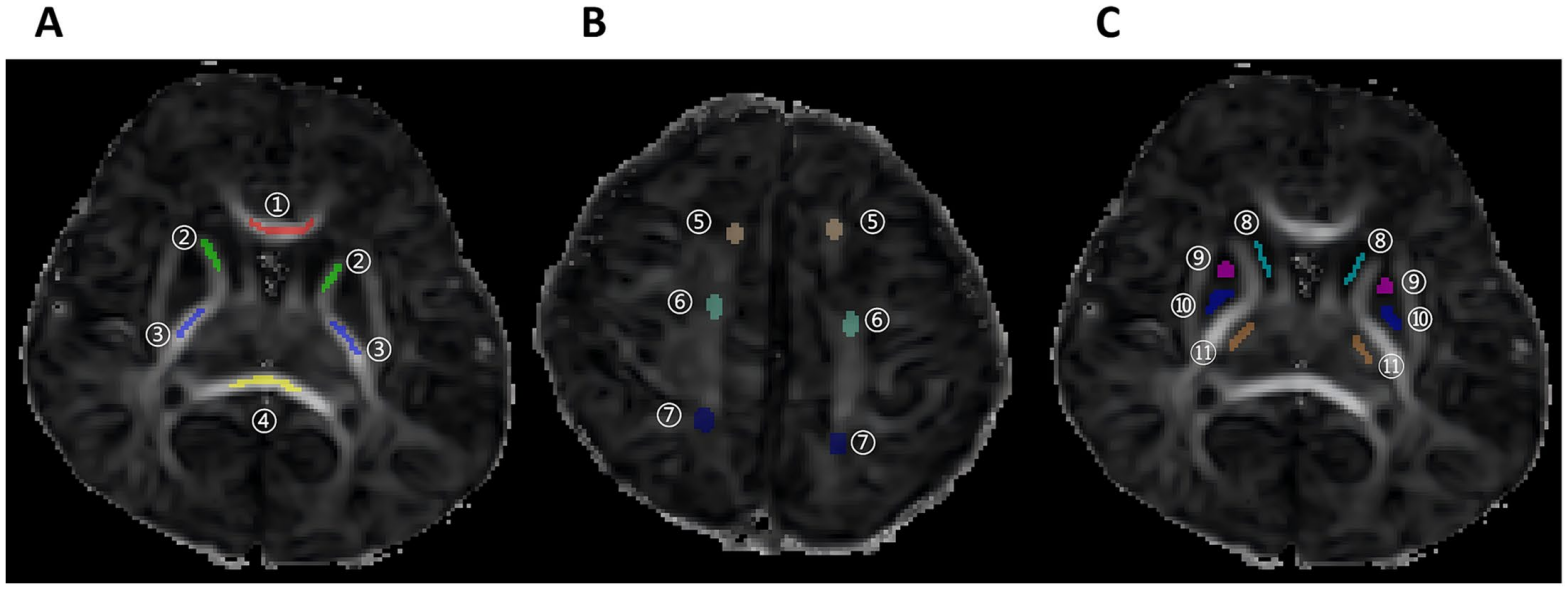
Schematic diagram of ROIs delineation. **(A)** Central white matter (level of the basal ganglia: ① genu of the corpus callosum [GCC], ② anterior limbs of internal capsule [ALIC], ③ posterior limbs of internal capsule [PLIC], and ④ splenium of the corpus callosum [SCC]); **(B)** peripheral white matter (level of the centrum semiovale: ⑤ frontal white matter [FWM], ⑥ central white matter [CWM] of the centrum semiovale level, and ⑦ parietal white matter [PWM]); **(C)** gray matter (level of the basal ganglia: ⑧ head of caudate nucleus [CN], ⑨ putamen [PUT], ⑩ globus pallidus [GP], and ⑪ thalamus [TH]).

ROIs were manually delineated using ITKSNAP (version 3.8.0) on 11 predefined white and deep gray matter regions (Figure 1), including the posterior limb of the internal capsule (PLIC), anterior limb of the internal capsule (ALIC), splenium of the corpus callosum (SCC), genu of the corpus callosum (GCC), frontal white matter (FWM), central white matter (CWM) at the centrum semiovale level, parietal white matter (PWM), head of the caudate nucleus (CN), globus pallidus (GP), putamen (PUT), and thalamus (TH). ROI placement was independently performed by a radiologist with 5 years of experience and reviewed by a senior neuroradiologist with over 10 years of experience. For all bilateral ROIs, the regions were delineated separately on the left and right sides, and the quantitative values of the left and right sides were averaged to obtain the final ROI parameter value for subsequent statistical analysis. Each ROI was measured three times, and mean values were used for subsequent analysis.

### 2.4 Apgar score assessment

Apgar scores (Pediatrics, 2015) were recorded at 1 and 5 min after birth for all neonates as part of routine clinical assessment. These scores were used to evaluate early postnatal condition and were included in correlation analyses with DKI-derived parameters.

### 2.5 Statistical analysis

Statistical analyses were performed using SPSS Statistics (version 21.0; IBM Corp.). Continuous variables are presented as mean ± standard deviation. Intergroup comparisons of DKI parameters were conducted using the Mann–Whitney U test. ROC curve analysis was applied to parameters showing significant group differences to assess diagnostic performance. Correlations between imaging parameters and Apgar scores were evaluated using Spearman rank correlation coefficients. *p* < 0.05 was considered statistically significant.

Given the retrospective and exploratory nature of this study, no *a priori* sample size calculation was performed, and no correction for multiple comparisons was applied. All eligible neonates meeting the inclusion criteria during the study period were included.

## 3 Results

### 3.1 Differences in DKI parameters between control and HIE groups

**Figure 2:**
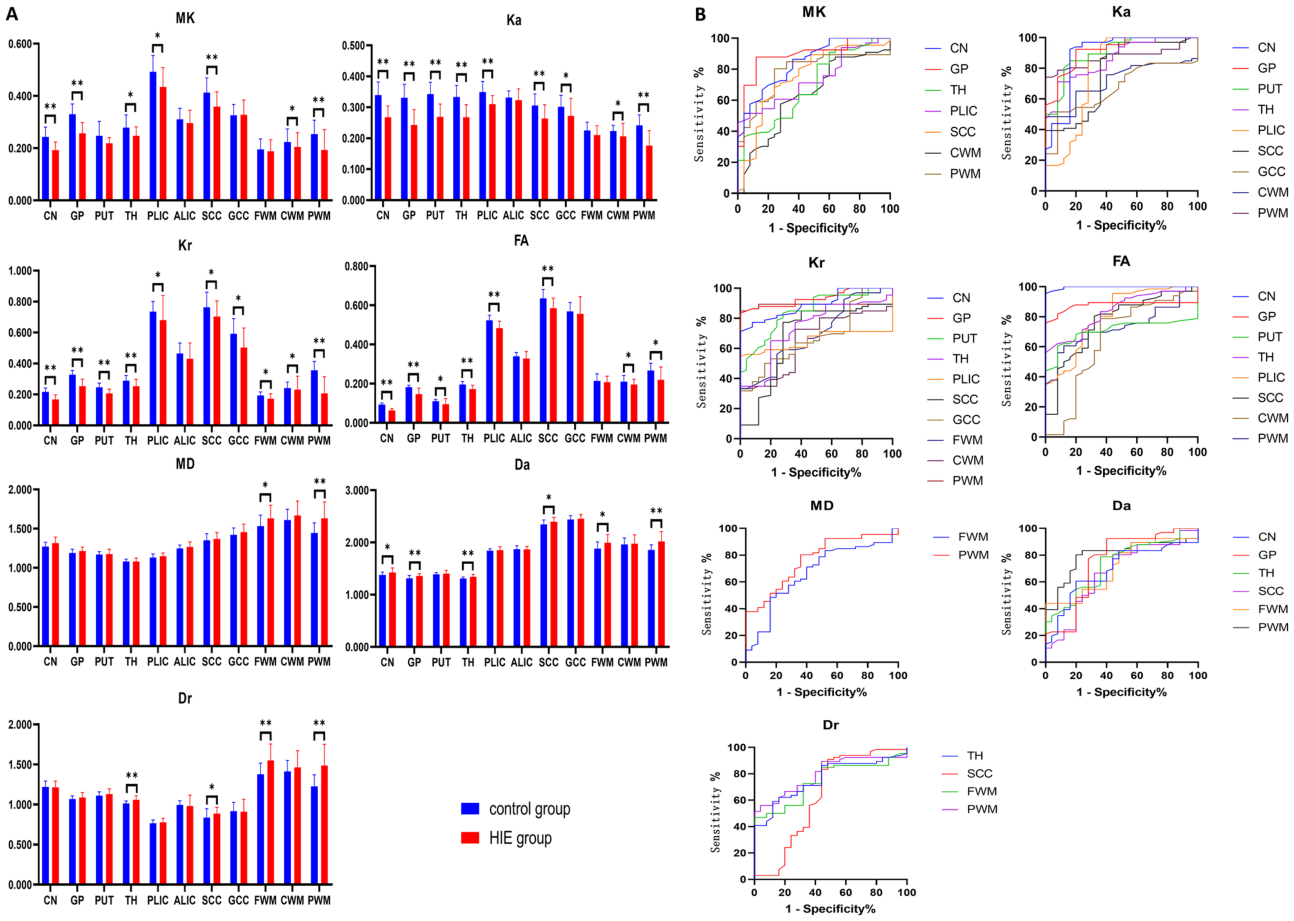
Comparison of DKI parameters and ROC analysis between the control group and HIE group. **(A)** Differences in DKI parameters between the control group and HIE group. **(B)** ROC analysis results for distinguishing the control group from the HIE group. The units of mean diffusivity (MD), axial diffusivity (Da), and radial diffusivity (Dr) are mm^2^/s. *indicates *p* < 0.05, and **indicates *p* < 0.001.

Group-wise comparisons of DKI parameters between control and HIE groups are shown in Figure 2A. Compared with controls, neonates with HIE exhibited widespread alterations in both kurtosis and diffusion metrics across deep gray matter and white matter regions, indicative of significant microstructural disruption following hypoxic–ischemic insult. Specifically, MK, Ka, and Kr values were significantly reduced in multiple regions, including the CN, GP, TH, PLIC, corpus callosum, and periventricular/central white matter. Furthermore, diffusivity measures demonstrated significant perturbations: FA values were reduced in several deep gray matter and white matter regions, while MD, Da, and Dr. values were predominantly increased in frontal and periventricular white matter regions.

ROC analyses of parameters exhibiting significant group differences are presented in Figure 2B, Supplementary Tables S1, S2. Several kurtosis metrics—particularly MK and Ka values within deep gray matter regions—demonstrated robust diagnostic performance, with area under the AUC values exceeding 0.800. Selected diffusion metrics, including regional FA and Da, also showed favorable discriminative accuracy, thereby supporting the utility of DKI as a sensitive imaging biomarker for the early detection of HIE.

### 3.2 Differences in DKI parameters across HIE severity subgroups

**Figure 3:**
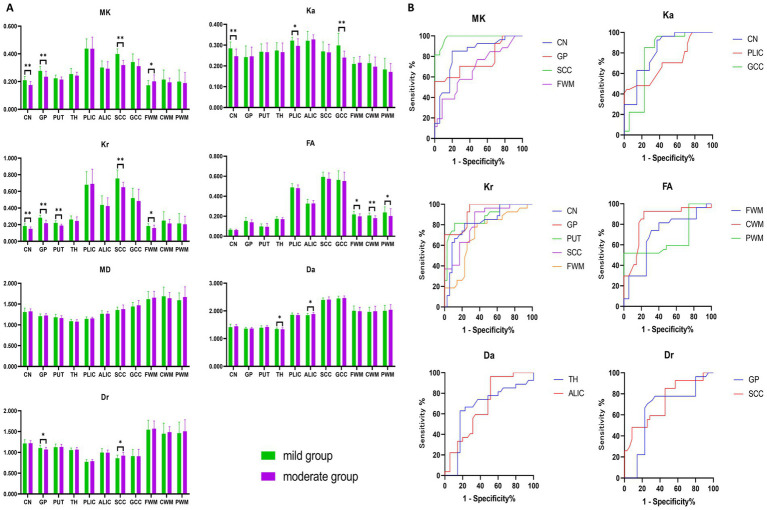
Comparison of DKI parameters and ROC analysis between the mild HIE group and moderate HIE group. **(A)** Differences in DKI parameters between the mild HIE group and moderate HIE group. **(B)** ROC analysis results for distinguishing the mild HIE group from the moderate HIE group. The units of mean diffusivity (MD), axial diffusivity (Da), and radial diffusivity (Dr) are mm2/s. *”indicates *p* < 0.05, and “**” indicates *p* < 0.001.

Comparisons between the mild and moderate HIE subgroups are presented in Figure 3A. Relative to the mild subgroup, the moderate subgroup demonstrated significantly lower MK values in the CN, GP, and SCC, whereas MK was elevated in the FWM. Ka values were significantly reduced in the CN, PLIC, and GCC, while Kr values decreased in the CN, GP, PUT, SCC, and FWM. Among diffusion metrics, FA was lower in the FWM, CWM, and PWM; Da was increased in the ALIC but reduced in the TH; and Dr. was elevated in the SCC but lower in the GP. No significant differences in MD were observed across all ROIs. ROC analyses (Figure 3B and Supplementary Table S3) showed that CN-MK, SCC-MK, and CWM-FA exhibited robust discriminative performance (AUC > 0.800), indicating that DKI can capture microstructural differences between mild and moderate HIE.

**Figure 4:**
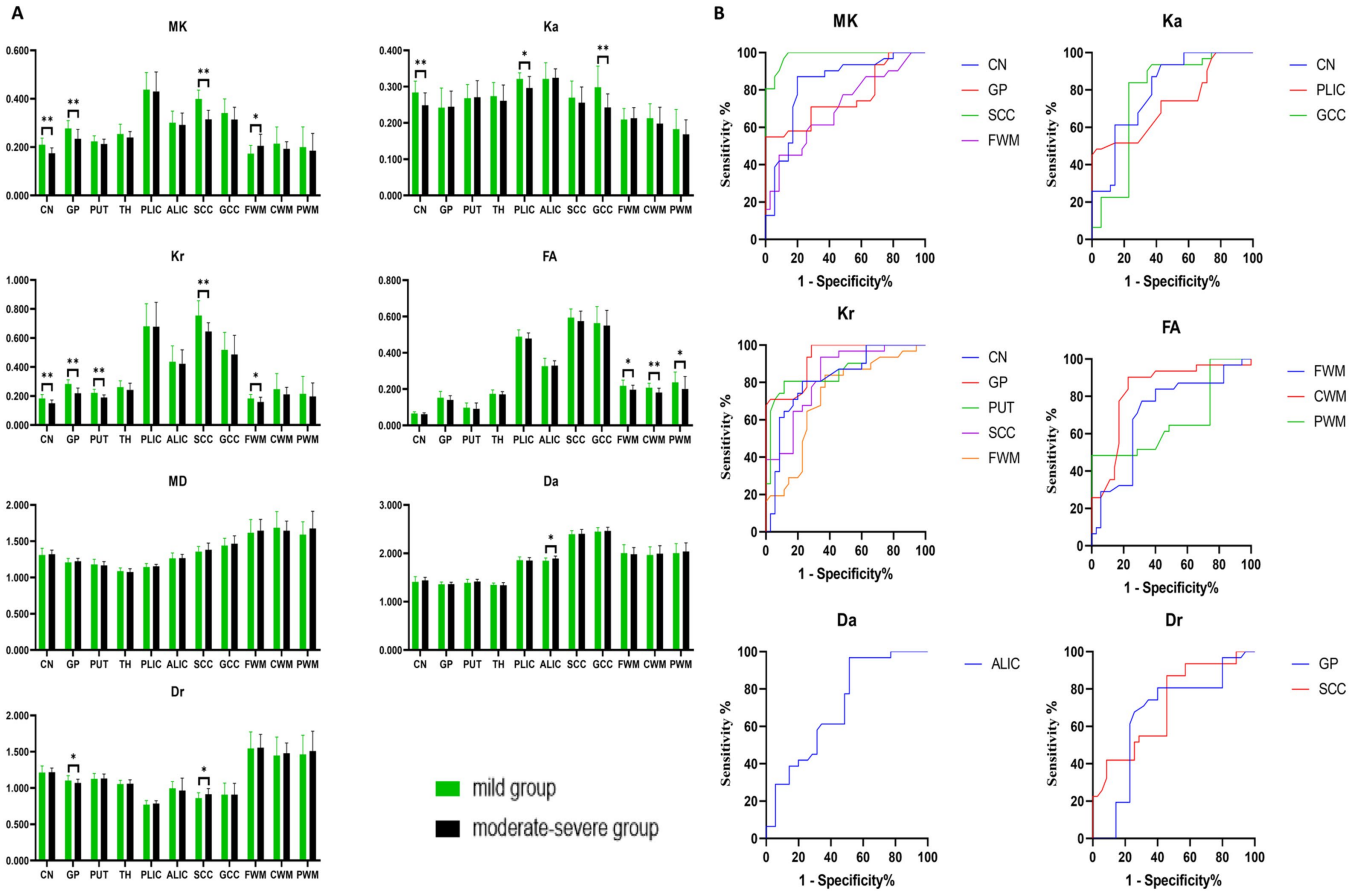
Comparison of DKI parameters and ROC analysis between the mild HIE group and moderate-to-severe HIE group. **(A)** Differences in DKI parameters between the mild HIE group and moderate-to-severe HIE group. **(B)** ROC analysis results for distinguishing the mild HIE group from the moderate-to-severe HIE group. The units of mean diffusivity (MD), axial diffusivity (Da), and radial diffusivity (Dr) are mm2/s. “*” indicates *p* < 0.05, and “**” indicates *p* < 0.001.

Given the limited number of severe cases (*n* = 4), the severe subgroup was merged into the moderate subgroup, and no separate severe subgroup was retained for analysis. The moderate-to-severe group comprised 31 cases (27 moderate + 4 severe). As shown in Figure 4A, compared with the mild subgroup, the moderate-to-severe subgroup exhibited reduced MK values in the CN, GP, and SCC, and increased MK in the FWM. Ka was lower in the CN, PLIC, and GCC, while Kr decreased in the CN, GP, PUT, SCC, and FWM. Diffusion metrics showed reduced FA in the FWM, CWM, and PWM, increased Da and Dr. in the SCC, and lower Dr. in the GP. ROC analyses (Figure 4B and Supplementary Table S4) confirmed that several kurtosis parameters and CWM-FA retained strong discriminative performance (AUC > 0.800), supporting the overall utility of DKI for stratifying HIE severity in term neonates.

### 3.3 Apgar scores and correlations with DKI parameters

Both 1-min and 5-min Apgar scores—a core clinical indicator for neonatal initial condition assessment in pediatric practice—were significantly lower in the HIE group than in the control group (both *p* < 0.001). Nevertheless, no significant variations in Apgar scores were detected among mild, moderate, and severe HIE subgroups, suggesting limited value of Apgar scores alone for stratifying HIE severity in term neonates.

To explore potential imaging biomarkers applicable to pediatric health monitoring of neonates with HIE, correlation analyses were performed focusing on DKI parameters that exhibited significant differences between the control and HIE groups (Tables 2, 3). Regarding kurtosis metrics, TH-Kr and PLIC-Kr were significantly correlated with 1-min Apgar scores, while TH-Kr, SCC-Kr, and PWM-Kr showed significant correlations with 5-min Apgar scores. For diffusion metrics, TH-Da correlated significantly with 1-min Apgar scores, whereas PUT-FA, TH-Da, and SCC-Da were significantly associated with 5-min Apgar scores, indicating DKI parameters may complement Apgar scores in evaluating neonatal neurological status post-hypoxic-ischemia.

**Table 2:** Correlation between 1-min and 5-min Apgar scores and DKI Kurtosis parameters.

ROIs	1-min Apgar score		5-min Apgar score	
	*r*	*p*	*r*	*p*
CN-MK	−0.137	0.273	−0.021	0.865
GP-MK	−0.237	0.055	−0.104	0.405
TH-MK	−0.100	0.423	−0.138	0.269
PLIC-MK	−0.189	0.129	−0.219	0.077
SCC-MK	0.207	0.096	0.233	0.060
CWM-MK	−0.004	0.978	−0.001	0.996
PWM-MK	−0.046	0.716	−0.206	0.098
CN-Ka	−0.177	0.155	0.015	0.905
GP-Ka	−0.169	0.176	0.072	0.565
PUT-Ka	−0.213	0.087	−0.076	0.546
TH-Ka	−0.166	0.184	−0.151	0.227
PLIC-Ka	0.075	0.549	0.161	0.197
SCC-Ka	0.152	0.224	0.095	0.448
GCC-Ka	0.139	0.265	0.125	0.318
CWM-Ka	0.125	0.316	0.152	0.224
PWM-Ka	0.226	0.069	0.050	0.689
CN-Kr	−0.11	0.927	−0.058	0.645
GP-Kr	−0.115	0.358	−0.137	0.271
PUT-Kr	−0.041	0.741	−0.072	0.564
TH-Kr	−0.313	0.010*	−0.351	0.004*
PLIC-Kr	−0.265	0.032*	−0.266	0.031
SCC-Kr	0.214	0.084	0.288	0.019*
GCC-Kr	−0.170	0.173	−0.099	0.431
FWM-Kr	−0.059	0.640	0.006	0.965
CWM-Kr	−0.145	0.245	−0.036	0.777
PWM-Kr	−0.137	0.274	−0.251	0.042*

“*r*” denotes the correlation coefficient; “–” indicates negative correlation; “*” signifies *p* < 0.05.

**Table 3:** Correlation between 1-min and 5-min Apgar scores and DKI diffusion parameters.

ROIs	1-min Apgar score		5-min Apgar score	
	*r*	*p*	*r*	*p*
CN-FA	−0.117	0.351	0.231	0.061
GP-FA	−0.102	0.415	0.063	0.615
PUT-FA	0.168	0.177	0.283	0.021*
TH-FA	0.025	0.841	0.073	0.561
PLIC-FA	0.009	0.940	0.070	0.577
SCC-FA	−0.070	0.578	−0.056	0.656
CWM-FA	0.044	0.725	0.180	0.148
PWM-FA	0.015	0.908	−0.041	0.745
FWM-MD	0.161	0.197	0.137	0.274
PWM-MD	0.057	0.647	0.149	0.232
CN-Da	0.021	0.870	−0.020	0.872
GP-Da	−0.218	0.079	−0.163	0.191
TH-Da	0.301	0.014*	0.258	0.036*
SCC-Da	0.120	0.337	0.342	0.005*
FWM-Da	0.206	0.096	0.195	0.117
PWM-Da	0.099	0.428	0.156	0.210
TH-Dr	0.162	0.193	−0.026	0.835
SCC-Dr	−0.023	0.855	0.076	0.545
FWM-Dr	0.129	0.301	0.165	0.185
PWM-Dr	0.097	0.439	0.159	0.201

“*r*” denotes the correlation coefficient; “–” indicates negative correlation; “*” signifies *p* < 0.05.

## 4 Discussion

Volpe et al. summarized MRI features of HIE and established associations between these findings and pathological subtypes (Volpe, 2012), noting that specific lesion patterns reflect imaging-pathological correlations useful for guiding diagnosis and treatment but cannot alone serve as a reliable criterion for HIE severity grading. This underscores the need for complementary imaging tools to improve early and accurate severity stratification— a critical requirement for informed clinical decision-making and optimal allocation of public health resources for at-risk neonates.

DKI extends the DTI framework by overcoming the limitations of Gaussian diffusion models, quantifying voxel-wise deviations from free diffusion and enabling sensitive detection of heterogeneity in water molecule movement within tissues (Jensen and Helpern, 2010; Marrale et al., 2016). Compared with conventional diffusion techniques, DKI more accurately captures tissue microstructural changes and allows concurrent derivation of kurtosis and tensor parameters (Jensen and Helpern, 2010; Xiao et al., 2020; Paydar et al., 2014; Umesh Rudrapatna et al., 2014; Guo et al., 2016). While DKI has been explored in brain tumors, traumatic brain injury, and stroke (Lesbats et al., 2020; Muftuler et al., 2020; Nagaraja, 2021), its clinical application in neonatal HIE— a leading cause of perinatal brain injury and long-term neurodevelopmental disability— remains relatively limited, as noted in the introduction. The present study used manual ROI delineation to compare DKI parameters between HIE neonates and healthy controls, and analyzed their associations with 1-min/5-min Apgar scores. Our findings confirm the significant advantages of DKI in diagnosing HIE and stratifying brain injury severity, which may support the optimization of neonatal public health surveillance systems targeting neurodevelopmental risk.

Comparisons between HIE and control groups identified significant differences in DKI parameters across deep gray matter, central white matter, and centrum semiovale regions. Among these, kurtosis parameters and FA values in deep gray matter and the centrum semiovale exhibited superior diagnostic efficacy, whereas the corpus callosum and anterior/posterior limbs of the internal capsule showed relatively weaker performance. This aligns with the primary injury pattern of HIE, where hypoxic–ischemic insults preferentially target watershed regions and metabolically active areas (Bano et al., 2017; Vermeulen et al., 2008)— a characteristic consistent with the pathophysiology of HIE described in the introduction. The basal ganglia, with high metabolic demand and blood flow requirements, and the centrum semiovale, a junction of deep perforating and cerebral cortical arterial supplies, thus display marked DKI parameter alterations and enhanced diagnostic utility. Notably, deep gray matter outperformed white matter in diagnostic performance: all deep gray matter regions achieved an AUC ≥ 0.900, with sensitivity and specificity both exceeding 80.0%. Tusor et al. similarly reported that deep gray matter damage is a common finding on conventional MRI in HIE (Tusor et al., 2012), and other studies have identified deep gray matter integrity as an independent predictor of HIE severity (Weeke et al., 2018). These collective findings confirm profound deep gray matter alterations following HIE and validate DKI’s potential as a scalable imaging biomarker for population-level neonatal HIE screening, particularly in resource-limited settings where identification of neurodevelopmental risk is critical for reducing long-term disability burdens.

In the comparison between mild and moderate HIE groups, CN-MK, SCC-MK, CN-Ka, CN-Kr, GP-Kr, PUT-Kr, SCC-Kr, and CWM-FA were significantly higher in the mild group, with all parameters showing diagnostic efficacies above 0.800. Among these, SCC-MK and GP-Kr performed best, with AUC values of 0.983 and 0.924, respectively. This aligns with Li et al.’s DTI study, which found that dense white matter regions such as the corpus callosum splenium and posterior limb of the internal capsule are particularly vulnerable in HIE, showing marked reductions in FA (Li et al., 2017). The corpus callosum is also rich in glutamate receptors, and excessive glutamate release following hypoxic–ischemic insult may induce excitotoxic damage, further increasing its susceptibility to injury (Kale et al., 2016). MK reflects overall tissue structural complexity, with higher values indicating greater diffusion restriction and more intricate microstructural organization. Kr, by contrast, reflects deviations in radial diffusion and is sensitive to oligodendrocyte and myelin integrity. Given that HIE preferentially impairs oligodendrocyte progenitor cells and disrupts myelination, and that the GP contains densely myelinated fibers, the pronounced changes in GP-Kr observed in our study are consistent with known patterns of injury in this region.

Although the severe HIE group included only four cases, we proceeded with subgroup analyses to explore the potential of DKI parameters in severity stratification. In mild versus severe comparisons, all significantly different parameters achieved diagnostic efficacies greater than 0.800. In moderate versus severe comparisons, only SCC-Ka reached statistical significance, with an AUC of 1.000. While this suggests that certain DKI parameters may distinguish severe HIE from other forms, the small number of severe cases may have influenced these results, highlighting the need for validation in larger cohorts. When moderate and severe cases were combined to mitigate sample size limitations, key parameters showed minimal differences compared with the mild group, with only TH-Da exhibiting notable variation. SCC-MK and GP-Kr retained high diagnostic performance, indicating that combining subgroups helped reduce the impact of small sample size on overall findings.

Collectively, our results show that MK and Kr differ significantly across HIE subgroups and are sensitive to both gray and white matter microstructural changes, supporting their utility in evaluating disease severity. Ka also differed in select comparisons and provides complementary information on fiber integrity and density. As a dimensionless measure, MK reflects overall structural complexity, while Kr and Ka capture directional aspects of diffusion, thereby addressing MK’s insensitivity to orientation. Together, these parameters enhance the ability of DKI to characterize the heterogeneous nature of HIE-related injury.

The Apgar score is a widely used, rapid indicator of neonatal condition at birth and is included in international diagnostic criteria for HIE (Azzopardi et al., 2008; Group of Neonatology, Chinese Pediatric Society, Chinese Medical Association, 2005). Consistent with previous studies showing strong associations between low Apgar scores and HIE (Lorain et al., 2022; Dalili et al., 2015), we found significant differences in both 1-min and 5-min scores between HIE and control groups (*p* < 0.001). Correlation analyses revealed significant associations between several DKI parameters and Apgar scores at both time points, suggesting that DKI may provide objective markers of perinatal hypoxic–ischemic status. More parameters correlated with 5-min Apgar scores, which may be explained by the fact that 1-min scores are influenced by transient factors such as maternal condition and delivery room management, and may not always reflect true hypoxic–ischemic injury (Lorain et al., 2022; Greenough et al., 1987). All correlation coefficients were weak (absolute values <0.400), likely due to the broad age range of included infants and the fact that Apgar scores assess acute perinatal status rather than long-term brain injury or prognosis (Pediatrics, 2015). Consequently, our analysis focused on the relationship between DKI parameters and Apgar scores as an initial exploration of potential links between imaging markers and acute clinical status.

Several limitations of this study should be noted. Several limitations should be acknowledged. First, the small sample size—particularly the severe HIE subgroup (*n* = 4)—and the single-center retrospective design limit the generalizability of our findings. Second, the 28-day MRI window may introduce temporal variability, although imaging timing did not differ significantly across groups (*p* = 0.123), and manual ROI delineation without formal ICC may introduce observer bias, though all ROIs were verified by a senior neuroradiologist. Third, the use of Apgar scores provides limited insight into long-term neurodevelopmental outcomes. Future multicenter prospective studies with larger cohorts, standardized imaging windows, automated segmentation, and long-term neurodevelopmental assessments are warranted.

## 5 Conclusion

This study confirms DKI’s utility in diagnosing neonatal HIE and stratifying severity. Kurtosis parameters and FA values differed significantly across groups, with deep gray matter outperforming white matter— a novel finding supporting its potential as a key imaging biomarker. DKI parameters correlated with 1-min/5-min Apgar scores, more strongly with the latter, providing objective microstructural evidence of perinatal hypoxic–ischemic status.

## Data Availability

The original contributions presented in the study are included in the article/Supplementary material, further inquiries can be directed to the corresponding authors.

## Footnotes

1
https://www.nitrc.org/projects/mricrogl/

2
https://fsl.fmrib.ox.ac.uk/fsl/fslwiki/

3
https://www.nitrc.org/projects/dke
