# Supplementary material for: Diagnostic value of diffusion kurtosis imaging for brain injury in term neonates with hypoxic–ischemic encephalopathy
Source: Front Neurosci. 2026 May 26;20:1806571. doi: 10.3389/fnins.2026.1806571 (PMC13246631; doi:10.3389/fnins.2026.1806571)
Supplement: Supplementary file 1 [file Table_1.DOCX]

Supplementary Material

**Supplementary Table 1.** ROC Analysis Results of Kurtosis Parameters in Control Group and HIE Group

| **ROIs-DKI** | **AUC** | **Youden Index** | **Sensitivity（%）** | **Specificity（%）** | **Cut-off value** |
| --- | --- | --- | --- | --- | --- |
| CN-MK | 0.844 | 0.507 | 66.7 | 84.0 | 0.212 |
| GP-MK | 0.891 | 0.759 | 87.9 | 88.0 | 0.293 |
| TH-MK | 0.691 | 0.324 | 36.4 | 96.0 | 0.224 |
| PLIC-MK | 0.735 | 0.455 | 45.5 | 100.0 | 0.404 |
| SCC-MK | 0.754 | 0.442 | 68.2 | 76.0 | 0.375 |
| CWM-MK | 0.634 | 0.296 | 57.58 | 72.0 | 0.196 |
| PWM-MK | 0.784 | 0.563 | 80.3 | 76.0 | 0.220 |
| CN-Ka | 0.893 | 0.764 | 92.4 | 84.0 | 0.310 |
| GP-Ka | 0.918 | 0.724 | 92.4 | 80.0 | 0.292 |
| PUT-Ka | 0.901 | 0.688 | 84.9 | 84.0 | 0.307 |
| TH-Ka | 0.862 | 0.632 | 71.21 | 92.0 | 0.297 |
| PLIC-Ka | 0.790 | 0.614 | 89.4 | 72.0 | 0.336 |
| SCC-Ka | 0.771 | 0.504 | 86.4 | 64.0 | 0.307 |
| GCC-Ka | 0.668 | 0.435 | 51.5 | 92.0 | 0.258 |
| CWM-Ka | 0.719 | 0.485 | 48.5 | 100.0 | 0.185 |
| PWM-Ka | 0.874 | 0.742 | 74.2 | 100.0 | 0.191 |
| CN-Kr | 0.892 | 0.712 | 71.2 | 100.0 | 0.185 |
| GP-Kr | 0.932 | 0.833 | 83.3 | 100.0 | 0.285 |
| PUT-Kr | 0.847 | 0.553 | 83.3 | 72.0 | 0.233 |
| TH-Kr | 0.737 | 0.452 | 65.2 | 80.0 | 0.264 |
| PLIC-Kr | 0.653 | 0.545 | 54.6 | 100.0 | 0.625 |
| SCC-Kr | 0.681 | 0.493 | 77.3 | 72.0 | 0.753 |
| GCC-Kr | 0.679 | 0.340 | 50.0 | 84.0 | 0.510 |
| FWM-Kr | 0.695 | 0.333 | 33.3 | 100.0 | 0.162 |
| CWM-Kr | 0.648 | 0.367 | 72.7 | 64.0 | 0.229 |
| PWM-Kr | 0.889 | 0.849 | 84.9 | 100.0 | 0.250 |

**Supplementary Table 2.** ROC Analysis Results of Diffusion Parameters in Control Group and HIE Group

| **ROIs-DKI** | **AUC** | **Youden Index** | **Sensitivity（%）** | **Specificity（%）** | **Cut-off value** |
| --- | --- | --- | --- | --- | --- |
| CN-FA | 0.997 | 0.955 | 95.5 | 100.0 | 0.079 |
| GP-FA | 0.878 | 0.758 | 75.8 | 100.0 | 0.163 |
| PUT-FA | 0.707 | 0.526 | 60.6 | 92.0 | 0.100 |
| TH-FA | 0.838 | 0.561 | 56.1 | 100.0 | 0.172 |
| PLIC-FA | 0.808 | 0.515 | 95.5 | 56.0 | 0.538 |
| SCC-FA | 0.764 | 0.483 | 80.3 | 68.0 | 0.626 |
| CWM-FA | 0.649 | 0.428 | 78.8 | 64.0 | 0.212 |
| PWM-FA | 0.728 | 0.486 | 60.6 | 88.0 | 0.225 |
| FWM-MD | 0.669 | 0.325 | 48.5 | 84.0 | 1.637 |
| PWM-MD | 0.767 | 0.443 | 80.3 | 64.0 | 1.464 |
| CN-Da | 0.691 | 0.406 | 60.6 | 80.0 | 1.413 |
| GP-Da | 0.752 | 0.524 | 92.4 | 60.0 | 1.311 |
| TH-Da | 0.733 | 0.428 | 78.8 | 64.0 | 1.310 |
| SCC-Da | 0.669 | 0.363 | 80.3 | 56.0 | 2.331 |
| FWM-Da | 0.714 | 0.439 | 43.9 | 100.0 | 2.076 |
| PWM-Da | 0.800 | 0.603 | 80.3 | 80.0 | 1.934 |
| TH-Dr | 0.763 | 0.471 | 59.1 | 88.0 | 1.053 |
| SCC-Dr | 0.639 | 0.429 | 90.9 | 52.0 | 0.805 |
| FWM-Dr | O.744 | 0.467 | 46.7 | 100.0 | 1.614 |
| PWM-Dr | 0.799 | 0.521 | 56.1 | 96.0 | 1.458 |

**Supplementary Table 3.** ROC Analysis Results of DKI-Derived Parameters in Mild and Moderate Groups

| **ROIs-DKI** | **AUC** | **Youden Index** | **Sensitivity（%）** | **Specificity（%）** | **Cut-off value** |
| --- | --- | --- | --- | --- | --- |
| CN-MK | 0.821 | 0.652 | 85.2 | 80.0 | 0.189 |
| GP-MK | 0.755 | 0.556 | 55.6 | 100.0 | 0.232 |
| SCC-MK | 0.983 | 0.857 | 100.0 | 85.7 | 0.368 |
| FWM-MK | 0.671 | 0.299 | 38.5 | 91.4 | 0.218 |
| CN-Ka | 0.817 | 0.554 | 92.6 | 62.9 | 0.282 |
| PLIC-Ka | 0.704 | 0.416 | 44.4 | 97.1 | 0.300 |
| GCC-Ka | 0.782 | 0.623 | 85.2 | 77.1 | 0.261 |
| CN-Kr | 0.816 | 0.586 | 81.5 | 77.1 | 0.171 |
| GP-Kr | 0.924 | 0.714 | 100.0 | 71.4 | 0.269 |
| PUT-Kr | 0.876 | 0.701 | 81.5 | 88.6 | 0.202 |
| SCC-Kr | 0.821 | 0.583 | 92.6 | 65.7 | 0.706 |
| FWM-Kr | 0.706 | 0.443 | 81.5 | 62.9 | 0.181 |
| FWM-FA | 0.693 | 0.426 | 74.1 | 68.6 | 0.200 |
| CWM-FA | 0.839 | 0.697 | 92.6 | 77.1 | 0.193 |
| PWM-FA | 0.664 | 0.519 | 51.9 | 100.0 | 0.168 |
| TH-Da | 0.662 | 0.458 | 63.0 | 82.9 | 1.320 |
| ALIC-Da | 0.683 | 0.449 | 96.3 | 48.6 | 1.841 |
| GP-Dr | 0.652 | 0.447 | 70.4 | 74.3 | 1.074 |
| SCC-Dr | 0.728 | 0.396 | 48.2 | 91.4 | 0.961 |

**Supplementary Table 4.** ROC analysis results of DKI-derived parameters in mild group and moderate-severe group

| **ROIs-DKI** | **AUC** | **Youden Index** | **Sensitivity（%）** | **Specificity（%）** | **Cut-off value** |
| --- | --- | --- | --- | --- | --- |
| CN-MK | 0.829 | 0.671 | 87.1 | 80.0 | 0.189 |
| GP-MK | 0.759 | 0.548 | 54.8 | 100.0 | 0.232 |
| SCC-MK | 0.983 | 0.857 | 100.0 | 85.7 | 0.368 |
| FWM-MK | 0.702 | 0.366 | 45.2 | 91.4 | 0.218 |
| CN-Ka | 0.801 | 0.507 | 93.6 | 57.1 | 0.285 |
| PLIC-Ka | 0.729 | 0.455 | 48.4 | 97.1 | 0.300 |
| GCC-Ka | 0.771 | 0.610 | 83.9 | 77.1 | 0.261 |
| CN-Kr | 0.818 | 0.578 | 80.7 | 77.1 | 0.171 |
| GP-Kr | 0.925 | 0.714 | 100.0 | 71.4 | 0.269 |
| PUT-Kr | 0.870 | 0.692 | 80.7 | 88.6 | 0.202 |
| SCC-Kr | 0.831 | 0.593 | 93.6 | 65.7 | 0.706 |
| FWM-Kr | 0.719 | 0.467 | 83.9 | 62.9 | 0.181 |
| FWM-FA | 0.711 | 0.460 | 77.4 | 68.6 | 0.200 |
| CWM-FA | 0.830 | 0.675 | 90.3 | 77.1 | 0.193 |
| PWM-FA | 0.670 | 0.484 | 48.4 | 100.0 | 0.168 |
| ALIC-Da | 0.704 | 0.453 | 96.8 | 48.6 | 1.841 |
| GP-Dr | 0.656 | 0.420 | 67.7 | 74.3 | 1.074 |
| SCC-Dr | 0.710 | 0.414 | 87.1 | 54.3 | 0.851 |
